# Liver Function Assessment in Oncology: Current Tools for Evaluating Hepatic Reserve and Surgical Decision-Making

**DOI:** 10.3390/jcm15208003

**Published:** 2026-10-15

**Authors:** Silvio Caringi, Antonella Delvecchio, Annachiara Casella, Valentina Ferraro, Matteo Stasi, Nunzio Tralli, Tommaso Maria Manzia, Riccardo Inchingolo, Michele Tedeschi, Riccardo Memeo

**Affiliations:** 1 Unit of Hepato-Biliary and Pancreatic Surgery, “F. Miulli” General Hospital, 70021 Acquaviva delle Fonti, Italy; a.delvecchio@miulli.it (A.D.); a.casella@miulli.it (A.C.); v.ferraro@miulli.it (V.F.); matteo.stasi@miulli.it (M.S.); n.tralli@miulli.it (N.T.); m.tedeschi@miulli.it (M.T.); r.memeo@miulli.it (R.M.); 2 Department of Medicine and Surgery, LUM University, 70010 Casamassima, Italy; 3 Transplant and HPB Unit, Department of Surgery Sciences, University of Rome Tor Vergata, 00133 Rome, Italy; manzia@med.uniroma2.it; 4 Unit of Interventional Radiology, “F. Miulli” General Hospital, 70021 Acquaviva delle Fonti, Italy; r.inchingolo@miulli.it

**Keywords:** liver function, future liver remnant, post-hepatectomy liver failure, liver resection, functional liver imaging, hepatobiliary scintigraphy, portal hypertension, hepatic functional reserve

## Abstract

Accurate assessment of hepatic functional reserve is essential in patients with primary or secondary liver malignancies undergoing liver resection, particularly in the presence of chronic liver disease, chemotherapy-associated liver injury, cholestasis, or portal hypertension. Conventional laboratory parameters and clinical scores, including Child-Pugh, MELD, ALBI, and the combined APRI/ALBI score, provide accessible measures of global hepatic status and risk stratification but do not directly quantify regional function or dynamic metabolic capacity. Complementary approaches include indocyanine green clearance, LiMAx testing, liver stiffness measurement, hepatobiliary scintigraphy, gadoxetic acid-enhanced magnetic resonance imaging, and CT/MRI-based future liver remnant volumetry. This narrative review critically examines the strengths, limitations, and clinical applicability of these modalities in the context of contemporary evidence and recommendations. Building on, but extending beyond, the 2023 E-AHPBA-ESSO-ESSR Innsbruck consensus, we incorporate subsequent comparative evidence on blood-based scores and functional imaging and discuss emerging approaches including longitudinal assessment, radiomics, and artificial intelligence. Current evidence supports an individualized, multimodal strategy in which volumetry remains central to surgical planning, while additional functional and portal-hypertension assessment is selected according to the extent of resection, underlying parenchymal disease, previous treatment, and local expertise. No single modality or fixed combination can currently be considered universally superior, highlighting the need for prospective validation of integrated and cost-effective assessment strategies.

## 1. Introduction

Assessment of hepatic reserve is essential when planning treatment for primary and secondary liver malignancies. The clinically relevant question is not simply whether conventional liver tests are within normal ranges, but whether the future liver remnant (FLR) will provide sufficient functional capacity after resection [1].

Routine laboratory tests and clinical scores remain useful first-line tools because they characterize hepatocellular injury, cholestasis, synthetic function, and chronic liver disease. However, they do not directly quantify the functional capacity of the planned remnant, a limitation that becomes particularly relevant before major hepatectomy and in patients with cirrhosis, steatosis, cholestasis, or chemotherapy-associated liver injury [1,2].

Contemporary preoperative assessment therefore combines clinical and laboratory risk stratification with cross-sectional imaging and FLR volumetry. In selected patients, additional evaluation may include liver stiffness measurement (LSM), indocyanine green (ICG) clearance, the Liver Maximum Capacity (LiMAx) test, hepatobiliary scintigraphy (HBS), gadoxetic acid-enhanced magnetic resonance imaging (MRI), or direct assessment of portal pressure [2].

The 2023 E-AHPBA-ESSO-ESSR Innsbruck consensus established the first European framework for individualized preoperative liver function assessment and emphasized FLR volumetry as the foundation of surgical planning, with additional testing selected according to the planned resection, underlying liver disease, portal-hypertension risk, previous systemic treatment, local expertise, and test availability [2].

Since that consensus, comparative studies have provided additional information on inexpensive blood-based scores and regional functional imaging. These data are clinically relevant because global, regional, static, dynamic, and hemodynamic tests address different components of hepatic reserve and should not be interpreted as interchangeable measures.

The purpose of this narrative review is to provide a clinically oriented synthesis of current methods for liver function assessment in patients undergoing oncologic liver surgery. Building on the 2023 Innsbruck consensus, the review incorporates subsequently published comparative evidence, critically examines blood-based, dynamic, clearance, hemodynamic, and imaging-based approaches, and discusses emerging strategies including longitudinal functional assessment, radiomics, and artificial intelligence. Particular emphasis is placed on how complementary tools may be integrated into individualized preoperative decision-making rather than considered as competing stand-alone tests.

## 2. Literature Review Approach

This article is a narrative, clinically oriented review rather than a systematic or scoping review. A targeted literature search of PubMed/MEDLINE, Scopus, and Web of Science was performed to identify relevant and current evidence on preoperative liver function assessment in adults undergoing liver resection. The search was last updated in August 2026.

Search terms included combinations of “liver function assessment”, “hepatic functional reserve”, “post-hepatectomy liver failure”, “future liver remnant”, “APRI ALBI”, “LiMAx”, “indocyanine green”, “liver stiffness”, “portal hypertension”, “HVPG”, “hepatobiliary scintigraphy”, “gadoxetic acid MRI”, “functional MRI”, “radiomics”, and “artificial intelligence”.

Priority was given to consensus statements and clinical guidelines, multicenter and comparative studies, systematic reviews, and clinically relevant original studies. Older landmark studies were retained when necessary to describe the development or validation of established tests. Additional references were identified from the bibliographies of key consensus documents and reviews.

Because the objective was narrative synthesis and clinical interpretation, no PRISMA-ScR flow diagram or formal study-level evidence charting was performed. The literature is discussed according to the principal assessment domains: blood-based and clinical scores, dynamic tests, clearance-based tests, imaging-based assessment, portal-hypertension evaluation, and emerging multimodal approaches.

## 3. Current Approaches to Liver Function Assessment

### 3.1. Static Biochemical Tests and Clinical Scores

Routine biochemical tests remain the most widely available first-line tools for assessing hepatic status because they are inexpensive, reproducible, and universally accessible. Serum bilirubin, albumin, transaminases, platelet count, prothrombin time, and the international normalized ratio (INR) provide information on hepatocellular injury, cholestasis, synthetic function, and indirect features of portal hypertension, but they do not directly measure hepatic functional reserve.

The Child-Pugh score combines biochemical variables (bilirubin, albumin, and coagulation) with clinical findings (ascites and hepatic encephalopathy). Although widely used in chronic liver disease, its subjective components and broad categories limit discrimination among patients with compensated disease [3].

The Model for End-stage Liver Disease (MELD) score is an objective laboratory-based prognostic tool developed for advanced liver disease. It was not designed specifically to predict post-hepatectomy liver failure (PHLF) after elective liver resection and may underestimate risk in patients with preserved conventional laboratory values but impaired parenchymal quality.

The Albumin-Bilirubin (ALBI) grade provides an objective estimate of hepatic reserve based on serum albumin and bilirubin and has demonstrated prognostic value in hepatocellular carcinoma (HCC) and liver resection [4,5]. More recently, the combined aspartate aminotransferase-to-platelet ratio index (APRI)/ALBI score has been proposed for preoperative risk stratification. In a large cohort, the combined score showed greater discrimination for grade C PHLF and liver dysfunction-related mortality than its individual components [6].

A European multicenter study subsequently compared APRI/ALBI with LiMAx and reported better discrimination for clinically significant PHLF with the blood-based score [7]. These findings are clinically relevant because APRI/ALBI is inexpensive and derived from routinely available laboratory parameters. However, interpretation should remain cautious because the comparison was retrospective and non-randomized, the population undergoing LiMAx testing was subject to selection effects, and the findings require independent external validation; methodological aspects of the comparison have also been debated in subsequent correspondence [7,8].

Portal hypertension and parenchymal stiffness provide additional information that is not captured by conventional liver scores. LSM should be considered in patients with suspected underlying liver disease, particularly those with HCC, although reported thresholds for postoperative risk remain heterogeneous [2]. Hepatic venous pressure gradient (HVPG) measurement can be considered selectively when cirrhosis with portal hypertension is suspected and non-invasive evaluation is insufficient. An HVPG of at least 10 mmHg is commonly used to define clinically significant portal hypertension, but this threshold is not universally applicable to all surgical subgroups [2]. Non-invasive findings such as thrombocytopenia, splenomegaly, portosystemic collaterals, and elastography can help determine which patients warrant further hemodynamic assessment.

Static blood tests and scores should therefore be interpreted as the first level of risk stratification, together with clinical findings, cross-sectional imaging, parenchymal quality, and portal-hypertension risk, rather than as direct measures of FLR function.

### 3.2. Dynamic Liver Function Tests

Dynamic liver function tests provide quantitative information on hepatic metabolic capacity that is not captured by conventional static biochemical parameters.

Among these techniques, LiMAx is one of the most extensively studied. After intravenous administration of 13C-methacetin, hepatic cytochrome P450 1A2 metabolism generates 13CO2, which is quantified in exhaled breath and used to estimate global hepatic metabolic capacity.

LiMAx has been evaluated as a quantitative marker for perioperative risk assessment, particularly when interpreted together with the planned extent of resection [9]. However, its incremental value is not uniform across patient populations, and comparative data indicate that readily available blood-based approaches such as APRI/ALBI may provide similar or greater discrimination in some settings [7].

Serial LiMAx measurements have also been explored after chemotherapy, portal vein embolization (PVE), and major liver resection [9]. Changes in measured function may be useful for longitudinal characterization, but improvement in a test value does not necessarily translate into improved prediction of PHLF. LiMAx should therefore be considered a complementary option rather than a mandatory or universally superior test.

Other breath-based approaches are being investigated as non-invasive measures of hepatic metabolic function. Among volatile organic compounds, exhaled limonene has received particular attention because impaired hepatic metabolism in cirrhosis is associated with reduced clearance and increased concentrations in exhaled breath. Clinical studies have demonstrated higher breath limonene concentrations in patients with cirrhosis than in healthy controls, with associations between limonene levels and conventional markers of hepatic dysfunction [10]. These approaches remain investigational and have not been validated for routine preoperative risk assessment before hepatectomy.

The main barriers to broader implementation of dynamic tests include cost, limited availability, the need for dedicated equipment, and incomplete standardization of clinically meaningful thresholds across different surgical populations.

Dynamic testing can therefore complement clinical, biochemical, and volumetric evaluation in selected patients, but current evidence does not support universal testing in every candidate for hepatectomy.

### 3.3. Clearance-Based Tests

Clearance-based tests quantify the hepatic uptake and/or elimination of exogenous compounds and provide a global estimate of functional capacity.

Indocyanine green (ICG) clearance is one of the most widely used quantitative tests in hepatobiliary surgery. ICG is taken up by hepatocytes and excreted into bile without metabolism. The principal clinical indices are the plasma disappearance rate (ICG-PDR) and the 15 min retention rate (ICG-R15).

ICG has been extensively evaluated for perioperative risk stratification, although its predictive performance varies across populations and clinical settings [11,12]. Consequently, fixed thresholds derived from selected cohorts should not be assumed to provide equivalent discrimination in all candidates for liver resection.

Combining ICG with FLR volumetry is conceptually attractive because global clearance and remnant volume provide complementary information. Nevertheless, the available evidence remains heterogeneous, and no universally accepted combined threshold has been established [11].

A major limitation of ICG is that it measures global rather than regional function. Its values are also influenced by hepatic perfusion, cardiac output, hyperbilirubinemia, and cholestasis. Therefore, a normal ICG result does not necessarily indicate adequate function of the planned FLR.

Other clearance-based methods, including galactose elimination capacity and monoethylglycinexylidide testing, have been investigated but are less commonly used in contemporary clinical practice.

ICG remains a useful quantitative tool in experienced centers, but its results should be interpreted together with FLR volume, parenchymal quality, and the overall clinical context [11].

### 3.4. Imaging-Based Functional Assessment

Imaging-based techniques complement conventional assessment by providing spatially resolved information on hepatic structure and, with selected tracers or contrast agents, regional function.

Hepatobiliary scintigraphy (HBS) using 99mTc-mebrofenin enables regional assessment of hepatocellular uptake and biliary excretion and can estimate functional FLR. However, comparative evidence does not establish universal superiority over anatomical volumetry. In a multicenter cohort of 547 patients undergoing major liver resection, remnant HBS function and remnant liver volume showed similar overall discrimination for PHLF, although HBS provided additional value in selected subgroups [13].

Gadoxetic acid-enhanced MRI combines high-resolution anatomical imaging with hepatocyte-specific contrast uptake. MRI-derived functional parameters have been associated with ICG clearance and postoperative outcomes [14,15]. In a 2025 multicenter study of 292 patients undergoing major hepatectomy, an MRI-derived hepatic uptake index improved identification of patients at risk of severe PHLF; importantly, discrimination was strongest when the imaging index was combined with MELD 3.0 rather than used alone [16].

These data support regional functional imaging as a complementary strategy when functional heterogeneity is expected, for example, after PVE, liver venous deprivation (LVD), associating liver partition and portal vein ligation for staged hepatectomy (ALPPS), in cholestasis, or before major hepatectomy. They do not demonstrate that functional imaging should replace volumetric analysis in all patients [13,16].

CT- or MRI-based FLR volumetry therefore remains the anatomical foundation of surgical planning. A standardized FLR of approximately 20% is commonly considered a minimum in patients with otherwise healthy liver parenchyma, whereas a larger reserve is generally required when parenchymal quality is impaired [2]. In cirrhosis, steatosis, cholestasis, or chemotherapy-associated injury, fixed volumetric thresholds are less informative and may be complemented by functional assessment.

Other imaging approaches, including CT perfusion, MR elastography, diffusion-weighted imaging, and hybrid PET/MRI, are under investigation for characterization of hepatic structure and function [17].

The broader use of functional imaging remains limited by cost, availability, dedicated software requirements, and incomplete standardization of acquisition protocols and clinically validated thresholds.

### 3.5. Emerging and Hybrid Approaches

An important contemporary trend is the integration of complementary measurements rather than reliance on a single test. PHLF is multifactorial, and volumetry, blood-based scores, clearance tests, dynamic metabolic tests, portal-hypertension markers, and regional imaging capture different components of hepatic reserve.

Accordingly, the incremental value of each modality depends on the patient population and clinical scenario. An individualized pathway should consider baseline clinical risk, FLR volume, underlying liver disease, previous systemic or locoregional treatment, portal-hypertension risk, and local availability of validated technologies.

Radiomics and artificial intelligence (AI) are emerging approaches that may extend information obtained from routine imaging. Radiomics extracts quantitative descriptors of texture, intensity, shape, and spatial heterogeneity that may reflect tissue characteristics not apparent on conventional visual assessment.

A systematic scoping review documented increasing use of deep learning and radiomics in liver imaging for fibrosis assessment, tumor characterization, functional evaluation, and outcome prediction [18]. More recent work has explored radiomics specifically for prediction of FLR volume and function before hepatic surgery [19].

Multi-omic approaches, including genomics, proteomics, metabolomics, and circulating biomarkers, are also being investigated as potential components of personalized risk assessment, although their role in routine preoperative liver surgery remains insufficiently established [20].

Longitudinal assessment represents another clinically relevant direction. Patients receiving neoadjuvant chemotherapy or undergoing PVE, LVD, or staged hepatectomy may experience substantial changes in liver volume and function between treatment steps. Repeated assessment may therefore provide information that a single preoperative measurement cannot capture.

Most emerging approaches remain limited by retrospective study designs, highly specialized study settings, heterogeneous acquisition and analysis protocols, and limited external validation. These limitations are particularly relevant to AI-based models, for which reproducibility and independent validation are prerequisites for clinical implementation.

Table 1 summarizes the principal assessment modalities, their clinical roles, strengths, limitations, and supporting references. A complementary overview of the parameters evaluated, advantages, limitations, and clinical applications of currently available liver function assessment methods is provided in Supplementary Table S1.

## 4. Discussion

Preoperative liver function assessment has evolved from isolated biochemical and volumetric measurements toward individualized multiparametric risk stratification. The clinically relevant distinction is not simply between ‘functional’ and ‘non-functional’ tests, but between modalities that characterize global hepatic status, regional FLR function, metabolic capacity, portal-hypertension-related risk, or anatomical reserve.

FLR volumetry remains the foundation of preoperative planning, particularly in patients with healthy parenchyma. Additional functional assessment becomes most relevant when liver volume may not accurately reflect reserve, including in cirrhosis, steatosis, cholestasis, chemotherapy-associated liver injury, or after liver augmentation procedures [21].

Blood-based assessment is no longer limited to Child-Pugh, MELD, and ALBI. The APRI/ALBI combination is attractive because it uses routinely available laboratory parameters and has demonstrated associations with severe PHLF and liver dysfunction-related mortality [6]. A multicenter comparison reported greater discrimination than LiMAx for clinically significant PHLF [7], but the retrospective design, selection of patients undergoing LiMAx testing, and subsequent methodological debate support cautious interpretation and independent validation [8].

LiMAx directly quantifies global metabolic capacity, but available evidence does not establish its superiority over other approaches in all patients. Its use may be most appropriate in selected high-risk patients or when serial measurements are clinically informative, provided local expertise and equipment are available [9].

Similarly, ICG clearance is reproducible and extensively used but reflects global function and is influenced by perfusion, bilirubin, and cholestasis. Its clinical interpretation is therefore strengthened when combined with information on FLR volume and parenchymal quality [11].

Regional functional imaging addresses hepatic heterogeneity but should not be described as universally superior to anatomical assessment. In the multicenter HBS study by Olthof et al., remnant liver function and remnant liver volume showed similar overall discrimination for PHLF [13]. Conversely, Bartholoma et al. demonstrated clinically useful discrimination with an MRI-derived hepatic uptake index, with the best performance obtained when the imaging parameter was combined with MELD 3.0 [16]. Together, these findings support integration rather than replacement of established assessment methods.

Portal-hypertension assessment is another important component of individualized risk evaluation. In patients with HCC or suspected chronic liver disease, LSM and non-invasive clinical or imaging markers can identify patients who may require more detailed evaluation; HVPG can be reserved for selected cases in which direct quantification of portal pressure may alter surgical decision-making [2].

Radiomics and AI may enable integration of imaging, clinical, and biochemical variables into more individualized risk models [18,19,22]. Automated FLR segmentation is a practical example of how deep learning may streamline preoperative planning [23].

Methodological limitations remain substantial. Many radiomics and machine-learning studies are retrospective and single-center, with heterogeneity in image acquisition, segmentation, feature extraction, and validation [18,19,22,23]. External validation, transparent reporting, and demonstration of incremental clinical value are required before routine implementation.

Longitudinal assessment may be particularly relevant during modern multimodal treatment. Changes in liver function during neoadjuvant chemotherapy, PVE, LVD, or ALPPS may not parallel changes in volume, and repeated measurements can potentially inform regeneration and timing of resection [21]. Prospective studies are needed to determine which tests provide clinically actionable information when repeated over time.

A pragmatic approach begins with clinical assessment, routine biochemical tests and scores including APRI/ALBI, cross-sectional imaging, and FLR volumetry. Patients with HCC or suspected chronic liver disease should undergo targeted evaluation for portal hypertension. Global functional tests such as ICG or LiMAx and regional tests such as HBS or gadoxetic acid-enhanced MRI can then be selected according to the planned resection, parenchymal quality, prior treatment, and the specific uncertainty that remains after baseline assessment.

**Figure 1:**
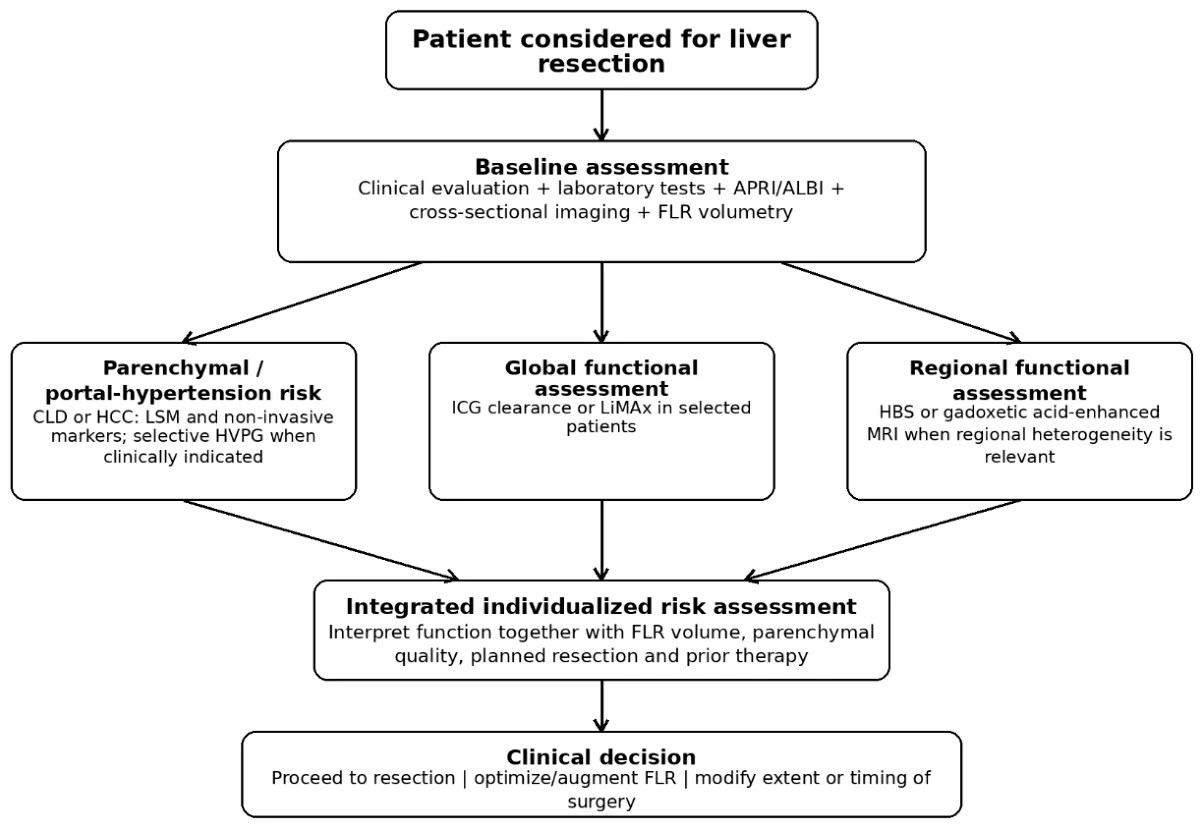
Conceptual framework for individualized preoperative assessment of hepatic reserve before liver resection. Baseline clinical, biochemical, and volumetric evaluation is complemented by assessment of portal hypertension and global or regional functional testing according to patient- and procedure-related risk. The framework represents the authors’ synthesis of the evidence discussed in this review. FLR, future liver remnant; APRI/ALBI, aspartate aminotransferase-to-platelet ratio index/albumin-bilirubin; CLD, chronic liver disease; HCC, hepatocellular carcinoma; LSM, liver stiffness measurement; HVPG, hepatic venous pressure gradient; ICG, indocyanine green; HBS, hepatobiliary scintigraphy; MRI, magnetic resonance imaging.

Figure 1 presents an original conceptual framework derived from the evidence synthesized in this review. Rather than reproducing a single published algorithm, it organizes preoperative assessment into baseline, portal-hypertension, global functional, and regional functional domains that converge on individualized clinical decision-making. A complementary stepwise algorithm illustrating the proposed assessment pathway according to the need for major hepatectomy, including functional evaluation, multidisciplinary review, and potential treatment strategies, is provided in Supplementary Figure S1.

## 5. Limitations

Several limitations of the available evidence should be acknowledged. Definitions of PHLF, patient-selection criteria, test thresholds, and acquisition protocols vary substantially across studies. Many reports are retrospective and originate from high-volume specialist centers, limiting generalizability. The evidence supporting several assessment modalities remains low to moderate, and direct head-to-head comparisons are uncommon [2]. In addition, this article is a narrative review and was not designed to provide an exhaustive systematic synthesis or formal certainty-of-evidence assessment.

Future studies should prospectively evaluate integrated algorithms that combine laboratory markers, portal-hypertension assessment, volumetry, global or regional functional tests, and selected computational approaches. Particular priorities include independent external validation, demonstration of incremental value over standard assessment, and evaluation of cost-effectiveness.

## 6. Conclusions

Preoperative assessment of hepatic reserve should be individualized according to the planned resection, FLR volume, parenchymal quality, previous oncologic treatment, and portal-hypertension risk. Routine laboratory tests and scores such as APRI/ALBI provide accessible initial risk stratification, while volumetry remains the anatomical foundation of surgical planning.

ICG and LiMAx provide global quantitative information, whereas HBS and gadoxetic acid-enhanced MRI can characterize regional function. LSM and, in selected patients with suspected cirrhosis and portal hypertension, HVPG provide complementary information on hemodynamic risk.

Current evidence supports these modalities as complementary rather than competing tools. Regional functional imaging may be particularly useful in selected high-risk patients or when heterogeneous function is expected, but it has not been shown to be universally superior to FLR volumetry [13,16].

No single modality or fixed multimodal pathway can currently be considered a universal gold standard. Prospective multicenter studies should determine which combinations improve prediction of clinically relevant PHLF, establish externally validated thresholds, and identify cost-effective strategies for different surgical populations.

## Figures and Tables

**Table 1:** Principal modalities for preoperative liver function and hepatic reserve assessment.

Modality	Domain Assessed	Main Clinical Role	Main Strengths	Key Limitations	References
Child-Pugh/MELD/ALBI	Global static clinical/biochemical status	Baseline risk stratification	Widely available; inexpensive	Do not directly measure FLR function; limited regional information	[3,4,5]
APRI/ALBI	Composite blood-based risk	Preoperative PHLF and mortality risk stratification	Routine laboratory parameters; inexpensive	Retrospective evidence; independent validation required	[6,7,8]
LSM	Parenchymal stiffness/indirect portal-hypertension risk	Selected patients with HCC or suspected chronic liver disease	Non-invasive; widely applicable	Heterogeneous cut-offs; affected by disease context	[2]
HVPG	Portal pressure	Selected patients with suspected cirrhosis and portal hypertension	Direct hemodynamic assessment	Invasive; not routinely required; no universal surgical cut-off	[2]
LiMAx	Global metabolic capacity	Selected high-risk patients; serial functional assessment	Quantitative dynamic measurement	Cost, equipment and availability; uncertain incremental value	[7,9]
ICG clearance	Global uptake/excretory function	Quantitative preoperative functional assessment	Established; rapid; quantitative	Affected by perfusion, bilirubin and cholestasis; no regional information	[11,12]
HBS	Regional hepatobiliary function	Functional FLR assessment	Regional quantification	Limited availability and standardization; not consistently superior to volumetry	[13]
Gadoxetic acid-enhanced MRI	Regional hepatocyte uptake plus anatomy	Regional functional characterization	Combines high-resolution anatomy and function	Protocol heterogeneity; limited standardized thresholds	[14,15,16]
CT/MRI volumetry	Anatomical FLR	Foundation of resection planning	Standardized; widely available	Volume may not reflect function in diseased or heterogeneous parenchyma	[2,21]
Radiomics/AI	Computational multimodal prediction	Emerging individualized risk prediction and automated segmentation	Potential integration of imaging and clinical data	Predominantly retrospective evidence; standardization and external validation required	[18,19,22,23]

## Data Availability

No new data were created or analyzed in this study. Data sharing is not applicable to this article.

## Supplementary Materials

The following supporting information can be downloaded at https://www.mdpi.com/article/10.3390/jcm15208003/s1, Figure S1: Multimodal Algorithm for Preoperative Liver Function Assessment; Table S1: Comparison of currently available methods for liver function assessment.

## Abbreviations

ALBI	Albumin-Bilirubin
ALPPS	Associating Liver Partition and Portal vein Ligation for Staged Hepatectomy
BCLC	Barcelona Clinic Liver Cancer
EASL	European Association for the Study of the Liver
FLR	Future Liver Remnant
Gd-EOB-DTPA	Gadoxetic acid
HCC	Hepatocellular Carcinoma
ICG	Indocyanine Green
ICG-PDR	ICG plasma disappearance rate
ICG-R15	ICG 15 min retention rate
LiMAx	Liver Maximum Capacity
LVD	Liver Venous Deprivation
MELD	Model for End-stage Liver Disease
PHLF	Post-Hepatectomy Liver Failure
APRI	Aspartate aminotransferase-to-platelet ratio index
HBS	Hepatobiliary scintigraphy
HVPG	Hepatic venous pressure gradient
LSM	Liver stiffness measurement
AI	Artificial intelligence
APRI/ALBI	Aspartate aminotransferase-to-platelet ratio index/Albumin-Bilirubin
CLD	Chronic liver disease
MRI	Magnetic resonance imaging
PVE	Portal vein embolization
