# Supplementary material for: Liver Function Assessment in Oncology: Current Tools for Evaluating Hepatic Reserve and Surgical Decision-Making
Source: J Clin Med. 2026 Oct 15;15(20):8003. doi: 10.3390/jcm15208003 (PMC13706092; doi:10.3390/jcm15208003)
Supplement: Supplementary file 1 [file jcm-15-08003-s001.zip › jcm-4533262-supplementary.pdf]

METHOD	MAIN PARAMETER EVALUATED	ADVANTAGES	LIMITATIONS	CLINICAL APPLICATION
**BIOCHEMICAL TESTS**	Hepatocellular injury and synthetic function	Widely available, inexpensive, reproducible	Indirect assessment of liver function	Initial screening
**CHILD-PUGH SCORE**	Global hepatic reserve	Easy to calculate, widely validated	Includes subjective variables	Cirrhosis and HCC
**MELD SCORE**	Severity of liver dysfunction	Completely objective	Not designed to predict PHLF	Advanced liver disease
**ALBI SCORE**	Albumin and bilirubin	Objective, continuous evaluation	Does not evaluate metabolic function	HCC risk stratification
**LIMAX TEST**	Maximal metabolic capacity	Quantitative assessment of liver function	Limited availability and high cost	Major hepatectomy
**ICG CLEARANCE**	Hepatic uptake and biliary excretion	Well validated and widely used	Evaluates only global liver function	Preoperative risk assessment
**HEPATOBILIARY SCINTIGRAPHY**	Regional liver function	Quantifies FLR function	Limited availability	Major liver resection
**GD-EOB-DTPA MRI**	Morphological and functional assessment	Simultaneous anatomical and functional evaluation	Lack of standardization	Functional imaging
**RADIOMICS AND AI**	Multidimensional prediction models	Personalized risk stratification	Lack of external validation	Emerging applications

Table S1. Comparison of currently available methods for liver function assessment.

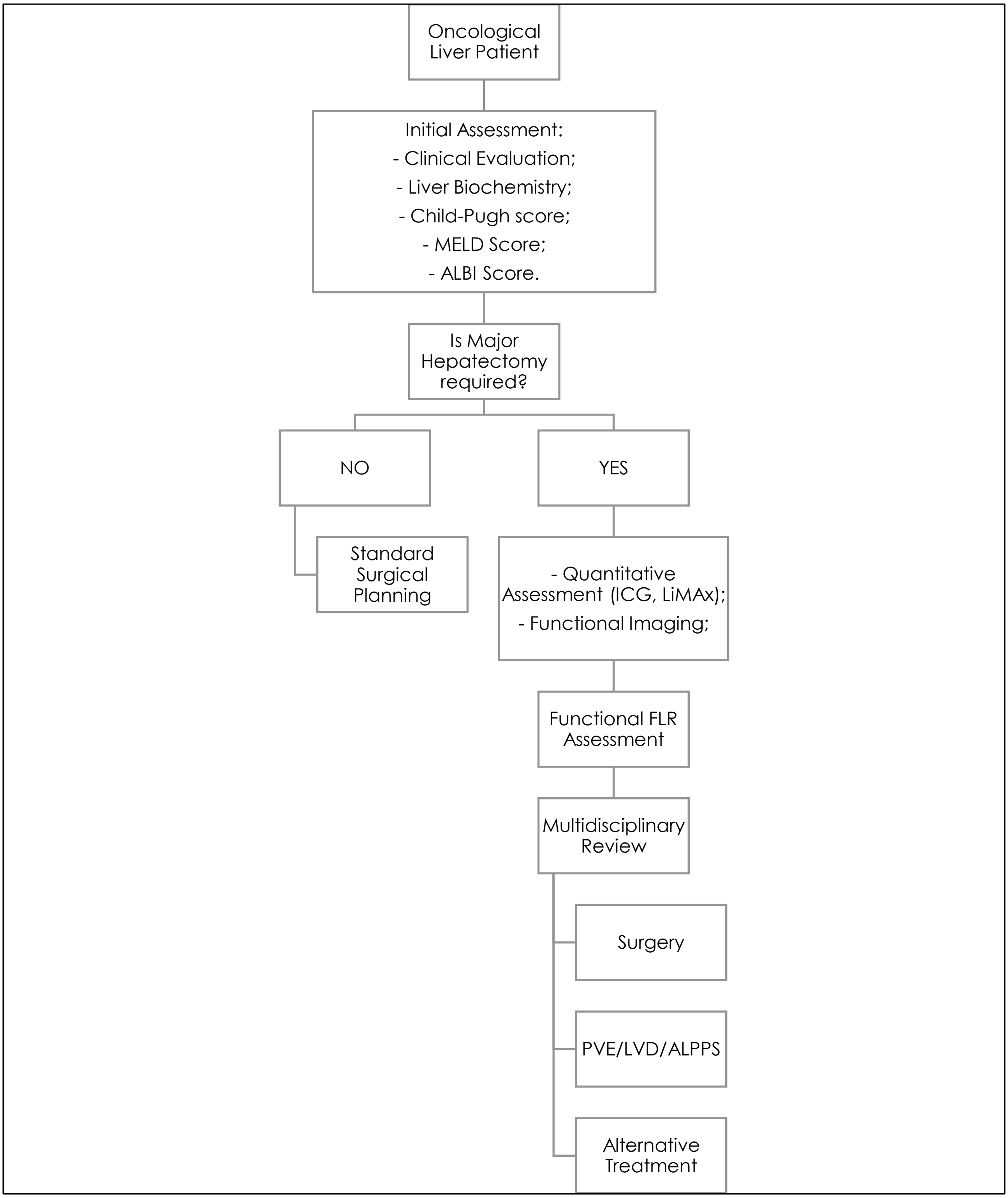

Figure S1. Multimodal Algorithm for Preoperative Liver Function Assessment.
